# Analysis of primary oral and oropharyngeal squamous cell carcinoma in inhabitants of Beijing, China—a 10-year continuous single-center study

**DOI:** 10.1186/s12903-020-01192-6

**Published:** 2020-07-17

**Authors:** Xue-Xiao Bai, Jie Zhang, Li Wei

**Affiliations:** 1 Department of Oral and Maxillofacial Surgery, Shenzhen People’s Hospital (The Second Clinical Medical College, Jinan University; The First Affiliated Hospital, Southern University of Science and Technology), Shenzhen, 518020 Guangdong China; 2 Department of Oral and Maxillofacial Surgery, Peking University School and Hospital of Stomatology, Beijing, 100081 China

**Keywords:** Prognostic factor, Survival rate, Oral squamous cell carcinoma, Beijing

## Abstract

**Background:**

This study aimed to describe some of the characteristics of the inhabitants of Beijing with oral and oropharyngeal squamous cell carcinoma (OSCC) who had received treatment at the Stomatology Hospital of Peking University and to analyze the survival rate and the prognostic factors of patients following surgical treatment.

**Methods:**

Data for 603 consecutive cases with primary OSCC between 2004 and 2013 were obtained from the Stomatology Hospital of Peking University. Clinical data including age, sex, living district, primary tumor site, TNM stage, history of drinking and smoking, pathological differentiation, treatment, and survival were retrospectively analyzed.

**Results:**

The male:female ratio was 1.1:1. The frequency of site involvement in descending order was tongue (34.3%), gingiva (25.0%), buccal mucosa (13.8%), oral floor (9.0%), oropharynx (8.5%), lip (6.3%) and palate (3.2%). Female OSCC patients tended to be significantly older than men with OSCC (*P* < 0.05). The percentage of patients with TNM stage III–IV OSCC was 52.4%. The results revealed that 65.3% of male patients and only 6.3% of female patients smoked or drank. The overall 5-year survival rate was 64%. Female patients had a worse prognosis than male patients. Among the patients who received surgery, tumor size and lymph node involvement were independent prognostic factors. Smoking and drinking were not prognostic factors.

**Conclusion:**

Among the Beijing inhabitants who were diagnosed with OSCC and treated in our hospital in the past 10 years, more than half were in the advanced stage, and the cancer stage was the main prognostic factor of oral cancer. Therefore, the recognition of oral cancer should be strengthened, and early detection and treatment of OSCC should be achieved to improve the survival rate.

## Background

Oral and oropharyngeal cancer is the term used to describe cancers that form in tissues of the oral cavity and the oropharynx. It is estimated that 354,864 cases of oral cavity carcinoma and 92,887 cases of oropharyngeal carcinoma were newly diagnosed during 2018, these cancers are ranked 18th and 26th among the most commonly encountered cancer sites, and approximately 228,389 patients died of these cancers [1]. Squamous cell carcinoma constitutes 90% of all oral malignancies [2]. Globally, there are geographical variations in the incidence of oral cancer. Cancers of the lip and oral cavity are very frequent in Southern Asia (e.g., India and Sri Lanka) as well as the Pacific Islands (Papua New Guinea has the highest incidence rate worldwide in both genders), and it is also the leading cause of cancer death among men in India and Sri Lanka [3].

In China, oral cancer is most prevalent in Hunan and Hainan provinces and in Taiwan, which could be attributed to the widespread use of betel nut. According to the National Cancer Center, cancer of the lip, oral cavity and pharynx (except the nasopharynx) is the 20th most common cancer in Beijing [4].

The Stomatology Hospital of Peking University is the largest center for OSCC in Beijing, and has the largest collection of cases. The present study aimed to describe some characteristics of Beijing inhabitants with OSCC who received treatment at the Stomatology Hospital of Peking University and to analyze the survival rate and the prognostic factors of these patients following surgery.

## Methods

### Study design and population

The data of 603 consecutive patients diagnosed with primary OSCC between 2004 and 2013 were obtained from the registration center of patients admitted to the Stomatology Hospital of Peking University.

#### Inclusion and exclusion criteria

Patients were screened according to the following inclusion criteria: 1) the patients were inhabitants of Beijing and 2) their pathological results indicated squamous cell carcinoma. Patients whose complete clinical data were unavailable, and those with recurrent carcinoma were excluded from this study.

Follow up information was drawn from the Beijing Cancer Prevention and Treatment Institution. The first day after diagnosis was set as the start date.

The anatomical site locations for the included cases of oral cancer were the lip (10th edition of the International Classification of Diseases [ICD-10]: C00), mouth or oral cavity (C01–06), and oropharynx (C09–10).

We retrospectively analyzed the clinical data of the subjects, including age, sex, district of residence, primary tumor site, TNM stage, smoking and alcohol consumption history, tumor differentiation, treatment, and survival.

TNM staging is based on computed tomography/magnetic resonance imaging reports and postoperative pathological reports [5]. Tumor differentiation was based on the WHO histological grading system [6].

This study was approved by the Ethics Committee of Peking University Hospital of Stomatology. Permission was obtained to access the database used for this study.

This study was conducted from 2014 to 2016, in Beijing, China.

### Statistical analysis

Database tables were built using Excel, and all data were processed and analyzed with SPSS 26.0 for Mac.

Differences in age between male and female groups, and differences in age between male patients with and without oral habits were compared with a rank sum test (Mann-Whitney U method); “oral habits” refer to cigarette smoking and/or alcohol consumption.

Survival curves were drawn using the Kaplan-Meier method. Univariate Cox proportional hazards logistic regression analysis using the enter method was used to estimate the survival probabilities of patients who received surgery. All potentially significant factors derived from univariate model (*P* < 0.05) were incorporated into multivariate Cox proportional hazards logistic regression analyses using the forward LR method. The hazard ratio (HR) and 95% confidence interval (CI) were calculated.

The significance test was two-sided. Goodness of fit was judged by the *P* value, and the significance level was set at 0.05.

## Results

### Gender and age

A total of 603 cases met the criteria for inclusion in this study, including 317 men and 286 women (male/female ratio 1.1:1). All descriptive variables are summarized in Table 1. The median age was 65 (56, 73) years. The median ages of the men and women were 63 (55, 72) and 67 (58.75, 74) years, respectively. Women were significantly older than men (*P* = 0.001). The age peak of the male patients with OSCC was 55–65 years, however, female OSCC patients had an age peak of 65–75 years. Of the 603 cases, 2.3 and 97.7% were < 40 and >  40 years, respectively (Table 1).

**Table 1:** Descriptive characteristics of OSCC

Variable	Category	N	%
Gender	Male	317	52.6
	Female	286	47.4
Age	≤ 40	14	2.3
	> 40	589	97.7
District	Urban	460	76.3
	Suburban	143	23.7
Tumor site	Tongue	207	34.3
	Gingiva	151	25.0
	Cheek	83	13.8
	Mouth floor	54	9.0
	Oropharynx	51	8.5
	Lip	38	6.3
	Palate	19	3.2
T stage	T1–2	397	65.8
	T3–4	206	34.2
Nodal metastasis	N0	394	65.3
	N+	209	34.7
pTNM stage	I	150	24.9
	II	137	22.7
	III	116	19.2
	IV	200	33.2
Tumor differentiation	I	210	46.3
	II	175	38.5
	III	22	4.8
	Unknown	47	10.4
Male	Tobacco	61	19.2
	Alcohol	9	2.8
	Both	137	43.2
	None	110	34.7
Female	Tobacco	13	4.5
	Alcohol	2	0.7
	Both	3	1.0
	None	268	93.7

### Anatomical sites and stage

The tongue was the most common site of oral cancer (34.3%), followed by gingiva (25.0%), buccal mucosa (13.8%), floor of the mouth (9.0%), oropharynx (8.5%), lip (6.3%) and palate (3.2%). OSCC of the mouth floor and oropharynx were significantly more common in male patients than in female patients: the respective male:female ratios were 6.7:1 and 2.4:1.

The percentages of patients staged as tumor size 1–2 and 3–4 were 65.8 and 34.2%, respectively; however, the percentage of TNM stage III–IV patients was 52.4%, indicating that more than half of the patients had late-stage OSCC (Table 1). The rate of lymph node metastasis was 34.7% and increased with tumor size, with the highest percentage of metastasis occurring in OSCC of the floor of the mouth (51.9%), followed by the oropharynx (especially at an early stage), gingiva, tongue, buccal mucosa, palate, and lip. Late stage cancer was more common in men (58.0%) than in women (46.1%).

### Oral habits

Our study found that 65.3% of male patients smoked and/or drank alcohol, whereas only 6.3% of female patients smoked and/or drank. The male patients who smoked or drank were significantly younger than those who neither smoked nor drank (Z = 6.089, *P* < 0.001).

### Prognosis

**Fig. 1:**
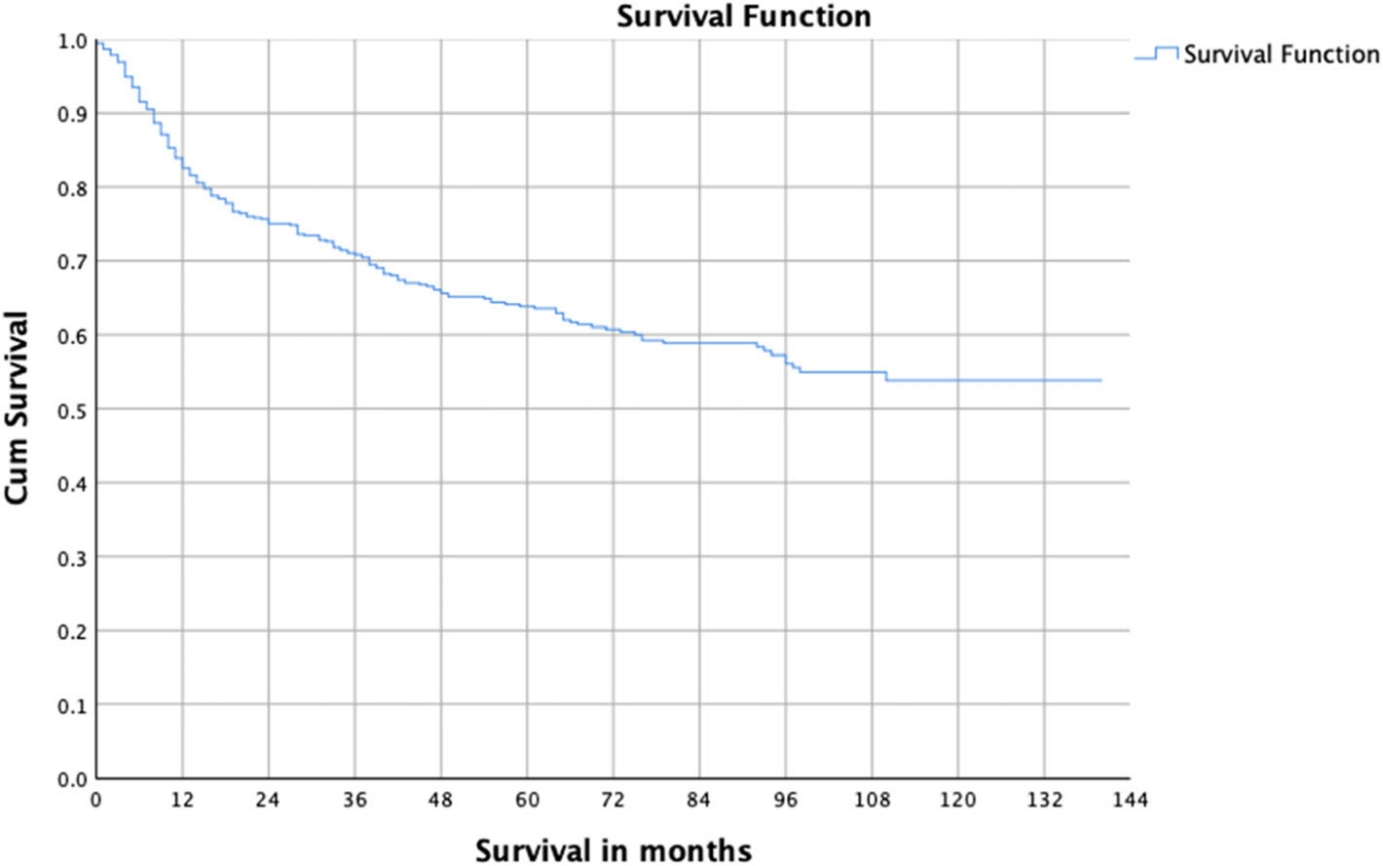
Survival curve of OSCC patients from 2004 to 2012

The follow-up duration was 10–140 months. The average follow-up time was 57.4 ± 37.3 months. The survival curve showed that survival decreased rapidly in the first 2 years after diagnosis, and then slowed down gradually thereafter; 3-, 5-, and 10-year survival rates were 71, 64 and 54%, respectively (Fig. 1).

### Prognostic factors of patients with surgery

**Fig. 2:**
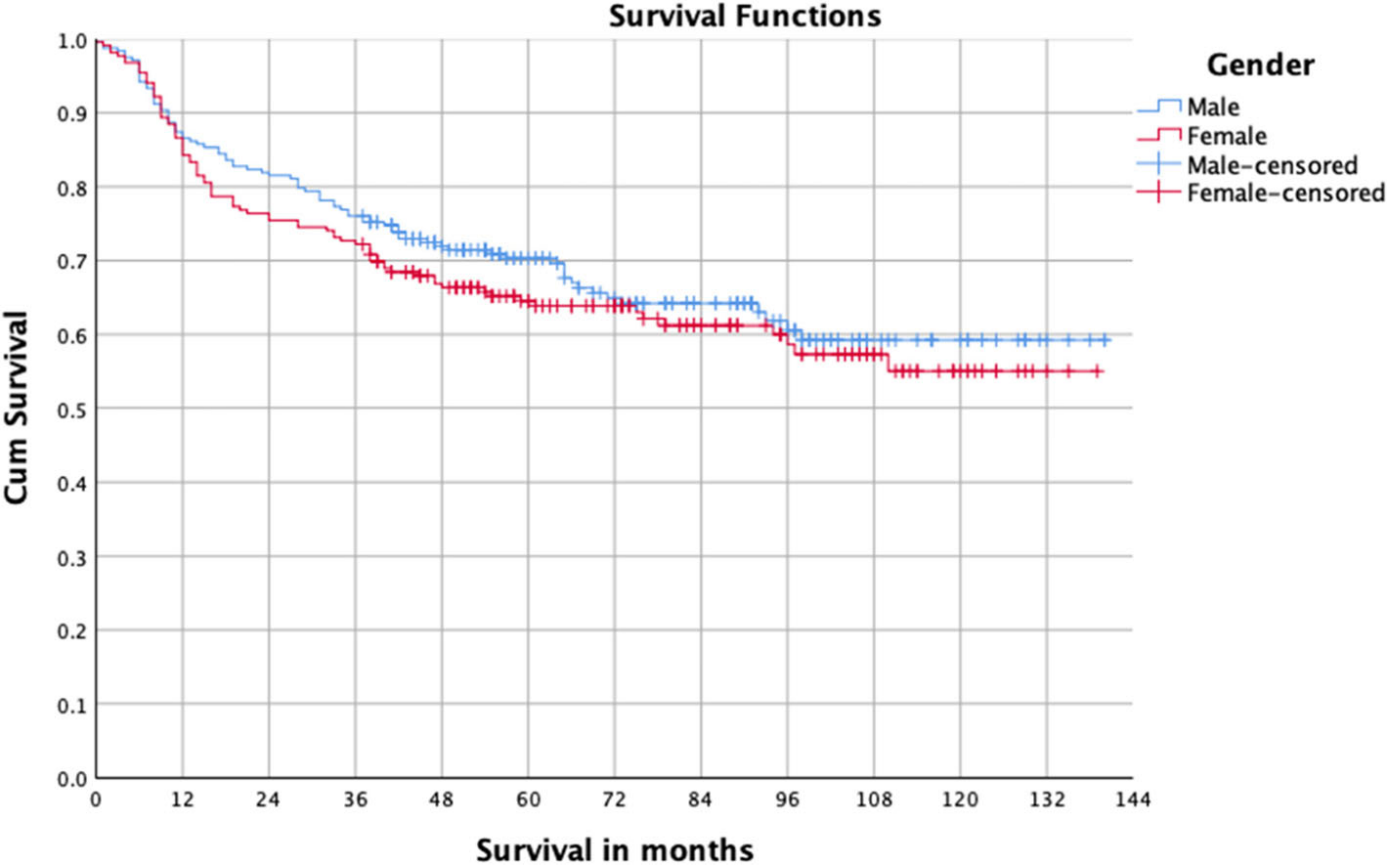
Survival curves of male and female OSCC patients

**Fig. 3:**
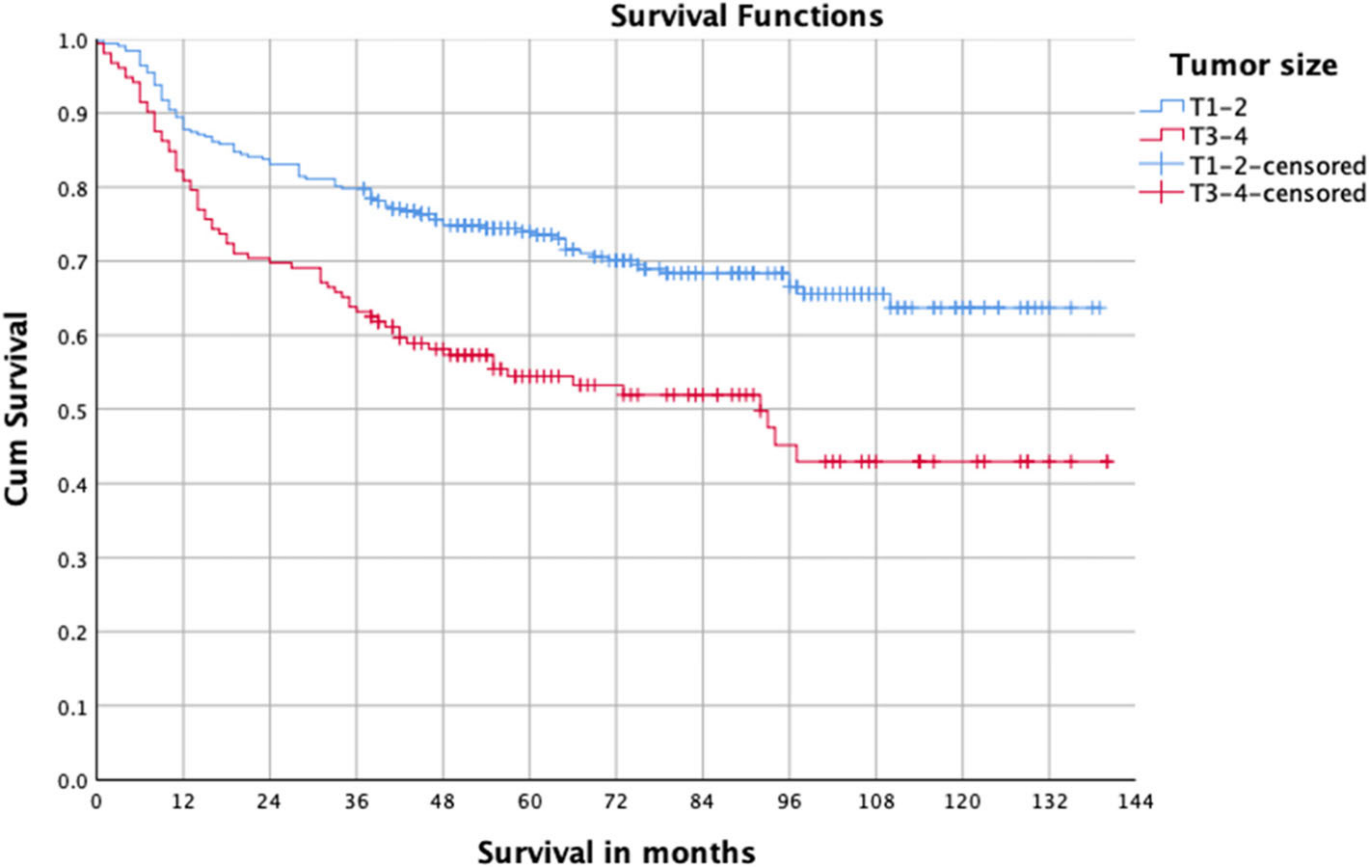
Survival curves of T1–2 and T3–4 OSCC patients

**Fig. 4:**
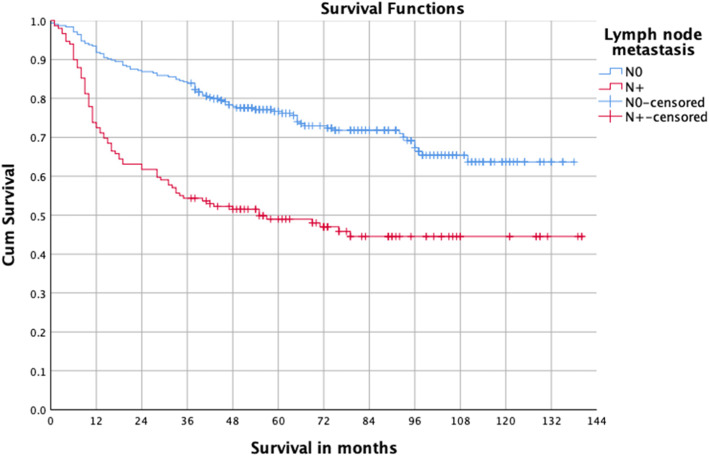
Survival curves of OSCC patients with and without nodal metastasis

Some patients cannot be treated surgically for a variety of reasons such as tumor invasion of important structures or serious cardiac disease. To better study the relationship between the surgery and prognosis, we conducted a survival analysis of patients with OSCC after undergoing surgical treatment. A total of 454 patients underwent surgery, and the five-year survival was 67.5%. Univariate analysis indicated that stage III–IV, T3–4, lymph node metastasis, moderate and poor differentiation, and postoperative radiotherapy were the risk factors for prognosis (Table 2, Figs. 2, 3 and 4). Multivariate analysis showed T3–4 and lymph node metastasis were independent risk factors (Table 3). The survival difference between male and female OSCC patients was close to statistical significance (*P* = 0.076).

**Table 2:** Univariate survival analysis of OSCC with surgery ^a^

Variable	HR	95% CI	*P*
Gender			
Male (*n* = 238)	1.147	(0.846, 1.557)	0.377
Female (*n* = 216)	1		
Age			
> 50 (*n* = 391)	0.780	(0.516, 1.178)	0.238
≤ 50 (*n* = 63)	1		
Place			
Suburban (*n* = 113)	1.262	(0.898, 1.774)	0.180
Urban (*n* = 341)	1		
Tumor site			
Oropharynx (*n* = 39)	1.103	(0.648, 1.875)	0.718
Oral cavity, lip (*n* = 415)	1		
pTNM stage			
III–IV (*n* = 232)	2.458	(1.774, 3.406)	< 0.001
I–II (*n* = 222)	1		
T stage			
T3–4 (*n* = 152)	1.896	(1.393, 2.581)	< 0.001
T1–2 (*n* = 302)	1		
Nodal metastasis			
N+ (*n* = 149)	2.426	(1.786, 3.295)	< 0.001
N0 (*n* = 305)	1		
Tumor differentiation			
II–III (*n* = 197)	1.840	(1.319, 2.565)	< 0.001
I (*n* = 210)	1		
Cigarettes			
Yes (*n* = 160)	0.901	(0.652, 1.246)	0.528
No (*n* = 294)	1		
Alcohol			
Yes (*n* = 115)	0.892	(0.625, 1.274)	0.529
No (*n* = 339)	1		
Neck dissection			
Yes (*n* = 334)	1.309	(0.911, 1.882)	0.146
No (*n* = 119)	1		
Postoperative radiotherapy			
Yes (*n* = 136)	1.858	(1.360, 2.538)	< 0.001
No (*n* = 318)	1		

^a^Univariate Cox proportional hazards logistic regression

**Table 3:** Multivariate survival analysis for OSCC with surgery^a^

Variable	HR	95%CI	*P*
T3–4	1.822	(1.308, 2.538)	< 0.001
Nodal metastasis (+)	2.348	(1.685, 3.271)	< 0.001
Female	–	–	0.076

^a^Multivariate Cox proportional hazards logistic regression

## Discussion

The incidence and characteristics of oral cancer shows geographical variations, which occur due to socioeconomic conditions, smoking, alcohol consumption, and lifestyle habits. In this study, we described the characteristics of Beijing inhabitants with OSCC in our hospital.

This study showed the high proportion of female patients with OSCC. Previous reports in China showed that the male:female ratio ranged from 1.94–3.19:1 in different districts [7, 8, 9]. The male:female ratio has reduced from 6:1 in 1950 to approximately 2:1 at present [10]. Whereas, the male:female ratio was 1.1:1 in this study, which was very close to that reported by a retrospective study in Norway [11]. Fu et al. reported that the male:female ratio for oral cancer incidence across 10 years (2003–2012) in Shanghai City was 1.41:1 [3]. In recent years there has been a trend of increasing proportion of women with OSCC; this may be due to their growing consumption of tobacco and alcohol^2.^ However, of the female patients in this study, only 5.5% smoked and 0.7% drank, indicating the influence of other risk factors. Suba et al. reported that female oral cancer patients were significantly older at diagnosis than their male counterparts, and the ratio of non-smokers, non-drinkers among elderly female oral cancer cases was surprisingly high, which was in accordance with this study^12.^ Suba et al. put forward the estrogen deficiency hypothesis in 2007, stating that estrogen deficiency and elevated fasting glucose in postmenopausal women may increase their risk of oral cancer [12, 13]. According to a Beijing health report in 2013, the average life expectancies of men and women were 79 and 83 years, respectively. A longer life-expectancy for women suggests more years after menopause, a longer exposure to the risk of estrogen deficiency, and consequently a higher risk of OSCC. The estrogen deficiency hypothesis could explain the higher age peak of women with OSCC and the high ratio of non-smokers and non-drinkers among female OSCC.

OSCC mainly occurs in older people. The average age at the time of diagnosis is approximately 60 years [10]. Previous domestic studies indicated that the mean age of diagnosis was 54–60 years [8, 9]. In this study, the median ages of male and female patients were 63 and 66 years, respectively. The median age at the initial diagnosis of oral cancer in Shanghai was 64 years (males: 62 years; females: 69 years) [3]. At the time of diagnosis in this study, 2.3% patients were younger than 40 years, which was lower than what has been reported globally (5%) [10].

The cancer sites in this study in decreasing frequency of occurrence are as follows: tongue, gingiva, buccal mucosa, oral floor, oropharynx, lip and palate. This is similar to that of previous domestic reports [7–9]. A previous study utilizing the Surveillance, Epidemiology, and End Results (SEER) database included 20,647 patients with OSCC diagnosed between 1975 and 2013. In that study, floor of the mouth and oral tongue represented the most common disease subsites, followed in descending order by gums, retromolar trigone, buccal mucosa, and hard palate [14]. In regions of China, South East Asia, and India, however, carcinoma of the buccal mucosa is one of the most common forms of OSCC [15–17]^.^ The difference in the predominant sites of occurrence of cancers may largely be related to various habits prevalent in the respective regions, such as the incidence of OSCC of the buccal mucosa results from the endemic regional practice of chewing betel quid.

Oral cancer can be macroscopically observed and touched, unlike cancers at many other sites, and diagnostic methods are available for early detection. Despite this, most patients were advanced stage at diagnosis. Tsai et al. reported that of the 16,691 oral cancer patients, 67.2% were found to be in stage III or IV diseases when they searched for treatment [18]. In this study, more than half of the patients in the present study (52.4%) were at stage III and IV. Late stage diagnosis is often associated with low social-economic status.

OSCC is considered a cancer with a poor prognosis, since the 5 year survival rate is reported as 50–63% [19–21]. In the Southern region of Thailand, the 5 year survival rate was 24.1%, which was attributed to the advanced stage of the disease when the patients were diagnosed and to the type of treatment provided [22]. A study carried out in the Netherlands, from 1989 to 2011, demonstrated that patients diagnosed with oral and oropharyngeal SCC responded better to treatments, increasing the survival rate to 67 and 48%, respectively, in cases of oral cavity and oropharyngeal cancers [23]. Quinlan-Davidson et al. studied 289 patients with OSCC who were treated with surgery followed by postoperative intensity modulated radiotherapy and observed a 5-year overall survival of 57% [24]. Some authors reported that survival rates were better in more developed countries than in developing ones [25]. The 5-year survival (64.0%) in this study was similar to that of SEER (65.3%) [26]. The differences in the overall survival may be attributed to variabilities in stage distribution; site distribution; and other variables such as host characteristics, comorbidity, and treatment policy [27].

The prognosis of OSCC worsened with progressing TNM stage [28]. In a nationwide cohort study of 16,691 oral cancer patients who underwent treatment between 2004 and 2008 in Taiwan, Tsai et al. found that the survival rate of oral cancer patients was 82.3% for stage I, 72.6% for stage II, 62.3% for stage III, and 39.2% for stage IV^18^. Multivariate analysis in the present study showed T3–4 and lymph node metastasis were independent prognostic factors. Lymph node metastasis had more prognostic significance than tumor size.

The association between tumor differentiation and prognosis is controversial. Most studies regarded differentiation as prognostic factor [29, 30]. Some authors considered that differentiation exerted no influence on prognosis [31]. Univariate analysis of OSCC with surgery showed that moderate and poor differentiation were independent prognostic factors. Multivariate analysis indicated that differentiation was not an independent prognostic factor. The prognostic significance of tumor differentiation was weaker than that of TNM stage.

This study has several limitations that should be addressed. First, this study on oral cancer was conducted in a single hospital, with a sample size of 604 patients in 10 years, which is relatively small. Second, since human papillomavirus (HPV) testing for oropharyngeal cancer has not been conducted in our hospital for nearly two years, there is a lack of HPV data in previous patients. Our next move is to extend the study to all hospitals in Beijing, which will help us acquire a full knowledge of the characteristics of Beijing OSCC.

## Conclusion

Of the oral cancer patients in Beijing who had been diagnosed and treated in our hospital over the past 10 years, more than half were in the advanced stage, and cancer stage was the main prognostic factor of oral cancer. Therefore, oral cancer recognition should be improved, and early detection and treatment should be achieved to improve the survival rate of oral cancer.

## Data Availability

The datasets used and/or analyzed during the current study are available from the corresponding author on reasonable request.

## Abbreviations

OSCC: Oral squamous cell carcinoma
SEER: Surveillance, Epidemiology and End Results

## References

[1] Bray F, Ferlay J, Soerjomataram I, et al. Global cancer statistics 2018: GLOBOCAN estimates of incidence and mortality worldwide for 36 cancers in 185 countries [J]. CA Cancer J Clin. 2018;68:394–424. 10.3322/caac.21492 30207593

[2] Warnakulasuriya S (2009). Global epidemiology of oral and oropharyngeal cancer. Oral Oncol.

[3] Jin-Ye F, Chun-Xiao W, Chen-Ping Z (2018). Oral cancer incidence in Shanghai ---- a temporal trend analysis from 2003 to 2012[J]. Bmc Cancer.

[4] Chen W, Zheng R, Baade PD (2016). Cancer statistics in China, 2015[J]. CA Cancer J Clin.

[5] Edge SB, Byrd DR, Compton CC, Fritz AG, Greene FL (2010). Trotti Cancer Staging Handbook – AJCC Cancer Staging Manual (7th ed.).

[6] Barnes LL, Eveson JW, Reichart PADS (2005). Pathology and genetics of head and neck tumours.

[7] Ming-hui D, Han-jiang WU. Clinical analysis of 5443 malignant oral and maxillofacial tumors in Hunan area. China J Oral Maxillofacial Surg. 2010;8(2):144–8.

[8] Fu JY, Gao J, Zheng JW (2014). Descriptive analysis of oral squamous cell carcinoma incidence in south and East China. China J Oral and Maxillofac Surg.

[9] Dai XM, Liu H, Wen YM (2002). Analysis of oral squamous cell carcinoma incidence of 3436 cases. Chin J Clin Oncol.

[10] Benzian H, Williams D (2015). The challenge of oral disease: a call for global action [J]. The oral health atlas. 2nd ed.

[11] Rikardsen OG, Bjerkli IH, Uhlin-Hansen L (2014). Clinicopathological characteristics of oral squamous cell carcinoma in northern Norway: a retrospective study. Bmc Oral Health.

[12] Suba Z (2007). Gender-related hormonal risk factors for oral cancer [J]. Pathol Oncol Res.

[13] Suba Z (2009). Györgyi Maksa, Szilvia Mihályi, et al. role of hormonal risk factors in oral cancer development [J]. Orv Hetil.

[14] Farhood Z, Simpson M, Ward GM (2019). Does anatomic subsite influence oral cavity cancer mortality? A SEER database analysis [J]. Laryngoscope.

[15] Huang CH, Chu ST, Ger LP (2007). Clinicopathologic evaluation of prognostic factors for squamous cell carcinoma of the buccal mucosa. J Chin Med Assoc.

[16] Bobdey S, Sathwara J, Jain A (2018). Squamous cell carcinoma of buccal mucosa: an analysis of prognostic factors [J]. South Asian J Cancer.

[17] Sahu PK, Kumar S (2019). Epidemiological Aspects of Oral Cancer in North Indian Population. Indian J Otolaryngol Head Neck Surgery.

[18] Tsai WC, Kung PT, Wang ST (2015). Beneficial impact of multidisciplinary team management on the survival in different stages of oral cavity cancer patients: results of a nationwide cohort study in Taiwan [J]. Oral Oncol.

[19] Messadi DV (2013). Diagnostic aids for detection of oral precancerous conditions. Int J Oral Sci.

[20] Adami GR, Adami AJ (2012). Looking in the mouth for noninvasive gene expression-based methods to detect oral, oropharyngeal, and systemic cancer. ISRN Oncol.

[21] Mehrotra R, Sharma N, Umudum H, Ceyhan K, Rezanko T (2012). The role of cytopathology in diagnosing HPV induced oropharyngeal lesions. Diagn Cytopathol.

[22] Pruegsanusak K, Peeravut S, Leelamanit V, Sinkijcharoenchai W, Jongsatitpaiboon J, Phungrassami T (2012). Survival and prognostic factors of different sites of head and neck cancer: an analysis from Thailand. Asian Pac J Cancer Prev.

[23] Braakhuis BJ, Leemans CR, Visser O (2014). Incidence and survival trends of head and neck squamous cell carcinoma in the Netherlands between 1989 and 2011. Oral Oncol.

[24] Quinlan-Davidson SR, Mohamed ASR, Myers JN (2017). Outcomes of oral cavity cancer patients treated with surgery followed by postoperative intensity modulated radiation therapy [J]. Oral Oncol.

[25] Warnakulasuriya S (2010). Living with oral cancer: epidemiology with particular reference to prevalence and life-style changes that influence survival. Oral Oncol.

[26] Surveillance epidemiology and end results (SEER). SEER Cancer statistics review 2009-2015. National Cancer Institute. Available from: http://seer.cancer.gov/statfacts/html/oralcav.html. Accessed 10 Dec 2017.

[27] Zanoni DK, Montero PH, Migliacci JC (2019). Survival outcomes after treatment of cancer of the oral cavity (1985–2015)[J]. Oral Oncol.

[28] Kreppel M, Eich HT (2010). Alexander Kübler, et al. Prognostic value of the sixth edition of the UICC’s TNM classification and stage grouping for oral cancer [J]. J Surgical Oncol.

[29] Grimm M (2012). Prognostic value of clinicopathological parameters and outcome in 484 patients with oral squamous cell carcinoma: microvascular invasion (V+) is an independent prognostic factor for OSCC [J]. Clin Transl Oncol.

[30] Kosunen A, Ropponen K, Kellokoski J, Pukkila M, Virtaniemi J, Valtonen H (2004). Reduced expression of hyaluronan is a strong indicator of poor survival in oral squamous cell carci- Noma. Oral Oncol.

[31] Al-Rajhi N, Khafaga Y, El-Husseiny J, Saleem M, Mourad W, Al-Otieschan A (2000). Early stage carcinoma of oral tongue: prognostic factors for local control and survival. Oral Oncol.

